# Chronic heat stress induces renal fibrosis and mitochondrial dysfunction in laying hens

**DOI:** 10.1186/s40104-023-00878-5

**Published:** 2023-06-03

**Authors:** Fumika Nanto-Hara, Makoto Yamazaki, Hitoshi Murakami, Haruhiko Ohtsu

**Affiliations:** Division of Meat Animal and Poultry Research, Institute of Livestock and Grassland Science, National Agriculture and Food Research Organization (NILGS), 2 Ikenodai, Tsukuba, Ibaraki 305-0901 Japan

**Keywords:** cGAS-STING, Heat stress, Laying hens, Mitochondrial DNA, Renal fibrosis

## Abstract

**Background:**

Heat stress in laying hens negatively affects egg production and shell quality by disrupting the homeostasis of plasma calcium and phosphorus levels. Although the kidney plays an important role in calcium and phosphorus homeostasis, evidence regarding the effect of heat stress on renal injury in laying hens is yet to be elucidated. Therefore, the aim of this study was to evaluate the effects of chronic heat stress on renal damage in hens during laying periods.

**Methods:**

A total of 16 white-leghorn laying hens (32 weeks old) were randomly assigned to two groups (*n* = 8). One group was exposed to chronic heat stress (33 °C for 4 weeks), whereas the other group was maintained at 24 °C.

**Results:**

Chronic heat exposure significantly increased plasma creatinine and decreased plasma albumin levels (*P* < 0.05). Heat exposure also increased renal fibrosis and the transcription levels of fibrosis-related genes (*COLA1A1*, *αSMA*, and *TGF-β*) in the kidney. These results suggest that renal failure and fibrosis were induced by chronic heat exposure in laying hens. In addition, chronic heat exposure decreased ATP levels and mitochondrial DNA copy number (mtDNA-CN) in renal tissue, suggesting that renal mitochondrial dysfunction occurs under conditions of heat stress. Damaged mitochondria leak mtDNAs into the cytosol and mtDNA leakage may activate the cyclic GMP-AMP synthase (cGAS) stimulator of interferon genes (STING) signaling pathway. Our results showed that chronic heat exposure activated the cGAS-STING pathway as indicated by increased expression of *MDA5*, *STING*, *IRF7*, *MAVS*, and *NF-κB* levels. Furthermore, the expression of pro-inflammatory cytokines (*IL-12*) and chemokines (*CCL4* and *CCL20*) was upregulated in heat-stressed hens.

**Conclusions:**

These results suggest that chronic heat exposure induces renal fibrosis and mitochondrial damage in laying hens. Mitochondrial damage by heat stress may activate the mtDNA-cGAS-STING signaling and cause subsequent inflammation, which contributes to the progression of renal fibrosis and dysfunction.

## Background

Heat stress is one of the most deleterious environmental stressors affecting the poultry industry worldwide [1], because poultry is highly sensitive to heat stress and their ability to dissipate body heat is low [2]. Heat stress decreases the egg weight and shell quality in laying hens [3]. Moreover, exposure of laying hens to a high temperature causes a decrease in the plasma calcium and phosphorus levels [4], which are important minerals for laying hens that affect egg production and shell quality [5, 6]. The kidney plays an important role in maintaining calcium and phosphorus homeostasis, which is balanced by gastrointestinal absorption and renal excretion [7]. Therefore, low plasma calcium and phosphorus levels in hens exposed to chronic heat stress may be associated with renal dysfunction. However, there remains a lack of evidence demonstrating an effect of heat stress on renal damage in laying hens.

The kidney requires a large amount of energy because of the reabsorption of ultrafiltrate by the glomeruli to maintain homeostasis [8]; thus, mitochondria are enriched in renal tubular cells. Mitochondria are key organelles involved in various processes related to energy production from free radicals and signal transduction [9]. Mitochondrial dysfunction causes increased oxidative stress, depletion of ATP, and cell death [10], which are associated with various health issues, such as cancer [11], cardiovascular disease [12], Alzheimer’s disease [13], neurodegeneration [14], and aging [15]. Recent studies showed a pathogenic role for mitochondrial damage in the development and progression of kidney disease in humans and mice [16], indicating that mitochondrial homeostasis and biogenesis are essential for maintaining normal kidney function.

Mitochondrial DNA (mtDNA) is a non-nuclear double-stranded circular DNA without introns [17]. The mitochondrial DNA copy number (mtDNA-CN) is regarded as a biomarker of mitochondrial function [18, 19], and its alteration reflects mitochondrial biogenesis and function [20]. Under environmental stress conditions (e.g., hypoxia, heat exposure, and cold temperature), reactive oxygen species levels increase in the mitochondria, which may result in a significant decrease in the mtDNA-CN [21–23]. In fact, under long-term heat stress conditions, tissue ATP levels and mtDNA-CN were significantly decreased in the liver of broilers [24]. Since the kidney is a high-energy organ containing a large amount of mitochondria, we hypothesize that heat stress induces severe renal mitochondrial damage in hens during the laying period.

In pathological states of acute and chronic kidney disease, mtDNA stress may contribute to cyclic guanosine monophosphate-adenosine monophosphate synthase (cGAS) stimulator of interferon genes (STING) pathway activation and type I IFN responses [25, 26]. STING is a key adaptor in the cytosolic DNA-directed signaling pathway [27, 28] and its activity is associated with several inflammation-related diseases [29–31]. The leakage of mtDNA from damaged mitochondria into the cytosol is a key trigger for the activation of the STING signaling pathway [32]. In laying hens, the role of the mtDNA-cGAS-STING pathway in renal inflammation and/or fibrosis has not been clarified yet.

Therefore, the objective of this study was to clarify the effect of chronic heat stress on renal damage in laying hens, with a particular focus on the involvement of renal fibrosis and mitochondrial dysfunction.

## Material and methods

### Ethics statement

All procedures were approved by the Animal Care Committee of the Institute of Livestock and Grassland Science, National Agriculture and Food Research Organization (NARO), Japan (Approval number: 21C118ILGS).

### Birds

The experiment consisted of a 4-week preliminary breeding period for adaptation and a 4-week experimental period. During the preliminary breeding period, the egg production status of 96 hens was recorded, and 16 laying hens (32 weeks old, i.e., the peak time of egg laying) with approximately the same body weight (1,580 ± 30 g), feed consumption rate, and egg production performance (average laying rate: 99.1%) were selected. The laying hens were raised individually in wire-floored cages (measuring 33 cm × 45 cm × 40 cm, width × height × depth) and fed with a corn-soybean meal-based diet (containing 0.3% non-phytate P, 3.3% calcium, 500 IU/kg vitamin D, 2.8 Mcal/kg ME, and 15.5% crude protein: Table 1). The basal diet was designed to meet or exceed all nutrients requirements recommended by the Japanese feeding standard for poultry [33]. They had free access to feed and fresh water. Birds were randomly divided into two group (*n* = 8). One group was exposed to heat stress (33 °C for 4 weeks), whereas the other group was maintained at 24 °C. The light regimen was 15 L:9 D and the dark period was from 19:00 to 04:00. Egg production and egg weight of each laying hen were recorded daily. Laying rate, average egg weight, and daily egg production were calculated. Once a week, the eggs were collected to determine the breaking eggshell strength, eggshell thickness, and egg weight using a digital egg tester (Model DET6500, NABEL Co., Ltd., Kyoto, Japan). Body weight and feed intake were recorded once a week and on the final day of the experimental period. Blood collection was performed from the brachial vein. The samples were stored in microtubes and centrifuged under refrigeration. All birds were euthanized, and renal tissue samples were removed as quickly as possible.

**Table 1:** Composition of the basal diet

Ingredients, %	
Corn	67.23
Soybean meal	22.97
Vegetable oil	0.42
Calcium carbonate	7.86
Dibasic calcium phosphate hydrate	0.91
Sodium chloride	0.27
*DL*-Methionine	0.09
Vitamin mixture^1^	0.10
Mineral mixture^2^	0.10
Selenium	0.05
Calculated value	
Crude protein, %	15.50
Metabolizable energy, Mcal/kg	2.80
Non-phytate P, %	0.30
Calcium, %	3.30
Vitamin D, IU/kg	500.00

^1^Vitamin mixture provided the following (per kilogram of diet): vitamin A (from retinyl acetate) 10,000 IU; cholecalciferol, 500 IU; vitamin E (from *DL*-α-tocopheryl acetate), 15 IU; vitamin K (menadione sodium bisulfate), 0.8 mg; riboflavin, 7 mg; *D*-calcium pantothenate, 5 mg; nicotinic acid, 25 mg; choline chloride, 400 mg; pyridoxine hydrochloride, 3 mg; folic acid, 1.5 mg; thiamine mononitrate, 1.5 mg; biotin, 0.2 mg; vitamin B_12_ (cyanocobalamin), 10 μg

^2^Mineral mixture provided the following (per kilogram of diet): iron (FeSO_4_⋅7H_2_O), 80 mg; manganese (MnCO_3_⋅nH_2_O), 60 mg; zinc (ZnO), 40 mg; copper (CuSO_4_⋅5H_2_O), 8 mg; iodine (calcium iodate), 0.5 mg

### Plasma analysis

Plasma was separated and stored at −20 °C until assayed. The plasma levels of calcium, phosphate, blood urea nitrogen (BUN), creatine, and albumin were analyzed using the biochemical auto-analyzer, BioMajesty JCA-BM 8060 (JEOL Ltd., Tokyo, Japan).

### Histological analysis and fibrosis area analysis

Renal tissues were fixed in 4% paraformaldehyde, dehydrated in 70%, 80%, 90%, 95%, and 100% ethanol, and embedded in paraffin. Paraffin-embedded tissues were sectioned at 4 μm, rehydrated in a series of xylene and ethanol solutions, and then used for Masson Trichrome staining, which stains normal tissue regions red, nuclei black, and fibrosis regions blue. Observations of histopathological changes in kidney tissues were performed by light microscopy (Leica, Nusslock, Germany). Eight fields per sample were randomly selected for fibrotic area quantification using Image J software, version 6.0 (Media Cybernetics, Inc., Rockville, MD, USA). Images were acquired under identical conditions at the same magnification. The fibrotic area was expressed as the percentage of the captured image area.

### Total RNA isolation, cDNA synthesis, and real-time polymerase chain reaction (PCR)

Total RNA was extracted from kidney samples using the RNeasy Mini Kit (Qiagen, Venlo, Netherlands) following the manufacturer’s instructions. Complementary DNA (cDNA) was synthesized from 1 μg of total RNA using random primers (TOYOBO, Tokyo, Japan) and Rever Tra Ace (TOYOBO). Real-time PCR was performed to measure mRNA expression levels using a QuantStudio 5 Real-time PCR system (Applied Biosystems, Foster City, CA, USA) and THUNDERBIRD SYBR qPCR Master Mix (TOYOBO, Tokyo, Japan). The primer sequences for the target and reference genes are shown in Table 2. PCR primers for chicken interleukin (IL)-12 were purchased from Qiagen.

**Table 2:** Sequences of the primers used for quantitative real-time PCR

Gene^1^	Primer sequences^2^ (5'→3')	Accession no	Source or reference of primer sequences
*COL1A1*	F: ACCTCAGCAAGAACCCCAAG	XM_025144131.2	[34]
	R: CTCACCGCCGTACTCAAACT		
*COL1A2*	F: GCGGTTTCTACTGGATTGA	NM_001079714.2	[34]
	R: AGCGAGACGGCTTATTTG		
*αSMA*	F: AAGCACCACTGAATCCCAAAG	NM_001031229.1	[34]
	R: CCAGAGTCAAGCACAATCCCT		
*TGF-β*	F: GCAAACTGCGTCTGACCG	NM_001318456.1	[34]
	R: ACGAAGAAGATGCTGTGGC		
*MDA5*	F: CGAATGAAAACCTGGGACAG	AB371640	[35]
	R: TGGTTTTGCCACTGCCTGTA		
*STING*	F: CGGCTGTGACATCTGGGAT	KP893157	[35]
	R: CCCGAGTCAGGATGGTCTC		
*IRF7*	F: ACAACGCCAGGAAGGATGTC	NM_205372	[35]
	R: CCAGCAGCATGAACATGTGA		
*MAVS*	F: GAACGCAAACCACCTTCAAC	NM_001012893	[35]
	R: CCAGGAGCAGCACTCAAATC		
*IFN-β*	F: TTGCCCACAACAAGACGTGA	GU119897/AY974089	[35]
	R: GTGTGCGGTCAATCCAGTGT		
*IFN-γ*	F: GTCAAAGCCGCACATCAAAC	NM_205149.1	[35]
	R: GGCTTTGCGCTGGATTCTC		
*IL-1β*	F: GGCCTGAGTCATGCATCGTT	NM_204524.1	[35]
	R: ATAAATACCTCCACCCCGACAA		
*IL-8*	F: GGCTTGCTAGGGGAAATGA	AJ009800	[31]
	R: AGCTGACTCTGACTAGGAAACTGT		
*CCL2*	F: GGCAGACTACTACGAGACCAACAG	L34553	[31]
	R: ACGGCCCTTCCTGGTGAT		
*CCL4*	F: CTTCACCTACATCTCCCGGC	NM_001030360	[36]
	R: CTGTACCCAGTCGTTCTCGG		
*CCL20*	F: AGGCAGCGAAGGAGCAC	NM_204438	[36]
	R: GCAGAGAAGCCAAAATCAAAC		
*18S rRNA*	F: TCAGATACCGTCGTAGTTCC	HQ873432.1	[37]
	R: TTCCGTCAATTCCTTTAAGTT		

^1^*COL1A1* Collagen type I alpha 1, *COL1A2* Collagen type I alpha 2, *αSMA* α-smooth muscle actin, *TGF-β* Transforming growth factor-β, *MDA5* Melanoma differentiation-associated gene 5, *STING* Stimulator of interferon genes, *IRF7* Type I interferon regulatory factor 7, *MAVS* Mitochondrial antiviral signaling, *IFN-* Type I interferon-, *IL-* Interleukin-, *CCL* C–C motif chemokine ligands, *18S rRNA* 18S ribosomal RNA

^2^*F* Forward, *R* Reverse

### Determination of mtDNA relative expression and copy number

Mitochondrial DNA was extracted from kidney samples using the DNeasy blood and tissue kit (Qiagen, Venlo, Netherlands) following the manufacturer’s instructions. All steps were completed at room temperature and each sample was processed at one time. Relative expression of mtDNA was measured using a QuantStudio 5 Real-time PCR system (Applied Biosystems, Foster City, CA, USA) using THUNDERBIRD SYBR qPCR Master Mix (TOYOBO, Tokyo, Japan). The primer sequences for the target and reference genes are shown in Table 3. The mtDNA copy number was determined using the equation copies = 2^^^(−*Ct*_*mt*_)/(−*Ct*_*reference*_).

**Table 3:** Sequences of the primers used for mtDNA analysis

Gene^1^	Primer sequences^2^ (5'→3')	Accession no	Source or reference of primer sequences
*ND4*^3^	F: CGCAGGCTCCATACTACTCG	NC_040970.1	[38]
	R: TTAGGGCACCTCATAGGGCT		
*COX1*^3^	F: CCATACTACTTACCGACCGCAACC	NC_040970.1	[24]
	R: GTGTCTACGTCCATTCCGACTGTG		
*ATP6*^3^	F: ATTCTCAAGCCCCTGCCTAC	NC_053523.1	[24]
	R: TCAGAGTTGGATGGTGGAGAGG		
*ND6*^3^	F: TAACAACAAACCTCACCCAGCC	NC_053523.1	[24]
	R: GTGTGTCTTTTGCTCGGTTGGA		
*β-actin*^4^	F: ATCCGGACCCTCCATTGTC	NM_205518.1	[24]
	R: AGCCATGCCAATCTCGTCTT		

^1^*ND4* NADH dehydrogenase subunit 4, *COX1* Mitochondrial cytochrome c oxidase1, *ATP6* ATP synthase F0 subunit 6, *ND6* NADH dehydrogenase subunit 6

^2^*F* Forward, *R* Reverse

^3^Genes were used to amplify fragment of mitochondrial DNA

^4^Genes were used to amplify fragment of cDNA

### Tissue ATP contents

Cellular ATP in kidney tissue was extracted using the ATP assay kit (TA100, Toyo B Net, Tokyo, Japan). Briefly, small pieces of tissue (about 0.1 g) were washed once with PBS, resuspended in ATP extraction reagent, and centrifuged at 1,000 × *g* for 10 min. The supernatant was used for the ATP assay. The ATP level was quantitated using an ATP assay kit with luciferin and luciferase according to the manufacturer’s instructions.

### Statistical analysis

All data were analyzed using a Student’s *t*-test. The individual laying hen was the experimental unit. Data are shown as the mean ± SE. The results were considered significant at *P* ≤ 0.05 and a trend at 0.05 < *P* ≤ 0.1.

## Results

### Effects of chronic heat stress on laying performance and eggshell quality

As shown in Table 4, chronic heat exposure resulted in a lower laying rate (%), egg weight (g), and daily egg production (g/d) during the experimental period (*P* < 0.05). Heat exposure also reduced eggshell strength (kg/cm^2^), thickness (mm), and eggshell weight (g). The results indicate that heat stress was induced in this study. In addition, plasma calcium and phosphate concentration were decreased when the birds were exposed to high temperature compared with the control group.

**Table 4:** Laying performance and eggshell quality

Item	Control			Heat stress			*P-*value
Laying rate^a^, %	99.6 ± 0.4			85.3 ± 2.5			< 0.05
Average egg weight^a^, g	58.2 ± 1.0			54.4 ± 1.0			< 0.05
Daily egg production^a^, g/d	57.9 ± 1.1			46.5 ± 2.1			< 0.05
Eggshell strength^b^, kg/cm^2^	4.9 ± 0.1			4.2 ± 0.1			< 0.05
Eggshell thickness^b^, mm	0.43 ± 0.00			0.38 ± 0.01			< 0.05
Eggshell weight^b^, g	6.7 ± 0.1			5.7 ± 0.2			< 0.05
Plasma calcium^a^, mg/dL	2.8 ± 0.2			2.1 ± 0.2			< 0.05
Plasma phosphate^a^, mg/dL	2.3 ± 0.4			1.2 ± 0.2			< 0.05

Data are presented as mean ± SEM; *P* < 0.05 was considered statistically significant

^a^Laying performance and plasma contents refers to the average data of each group of hens (*n* = 8)

^b^Eggshell quality refers to the average data of each group of eggs (*n* = 8 for each week)

### Effect of chronic heat stress on renal function and histology

**Fig. 1:**
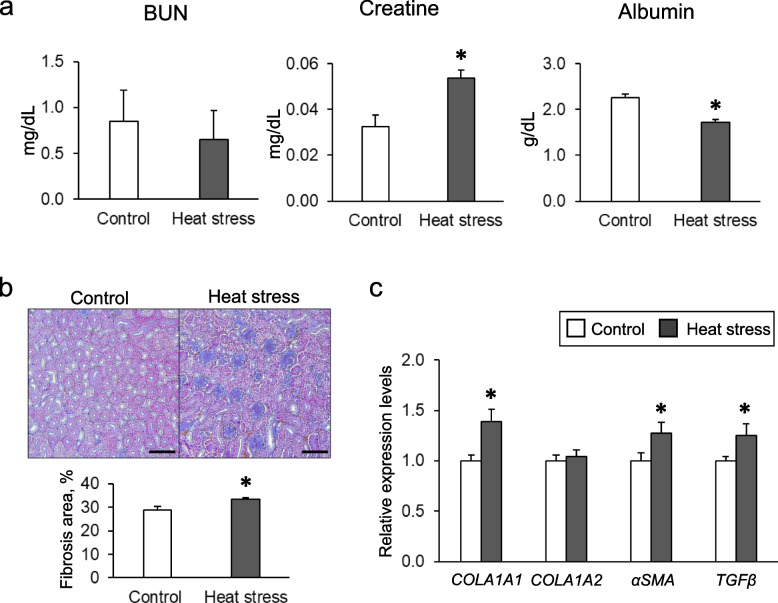
Effect of chronic heat stress on renal function and histology in laying hens. **a** Plasma levels of blood urea nitrogen (BUN), creatine, and albumin in the control (*n* = 8) or heat stress for 4 weeks (*n* = 8) hen groups. Data are presented as the mean ± SEM. Statistical analysis was performed by a Student’s *t*-test. ^*^P < 0.05 was considered statistically significant. **b** Representative histological images of Masson’s trichrome stained sections of the control and heat-stressed hen kidneys (upper). Morphometric analysis of the fractional cortical tubular area of Masson’s trichrome stained kidney images (lower). *n* = 8 for each group. Data are presented as the percentage of the total cortex and mean ± SEM. Statistical analysis was performed using a Student’s *t*-test. Bars = 100 µm. **c** The mRNA expression levels of *COL1A1*, *COL1A2*, *αSMA*, and *TGFβ* were measured by performing real-time PCR and normalized to 18S rRNA. *n* = 8 in each group. Statistical analysis was performed by a Student’s *t*-test

While the BUN in plasma was not affected adversely, chronic heat exposure significantly increased the level of plasma creatinine and decreased the level of plasma albumin, indicating renal dysfunction (Fig. 1a). As shown in Fig. 1b, Masson’s trichrome staining revealed that the renal fibrosis area was significantly increased in the heat-stressed group. Using quantitative PCR analysis, gene expression levels of collagen type I alpha 1 (*COL1A1*), α-smooth muscle actin (*αSMA*), and transforming growth factor-β (*TGF-β*) were increased in the heat-stressed group (Fig. 1c). These data suggest that heat exposure induces renal dysfunction and fibrosis in laying hens.

### Effect of chronic heat stress on mitochondrial function in the kidney

**Fig. 2:**
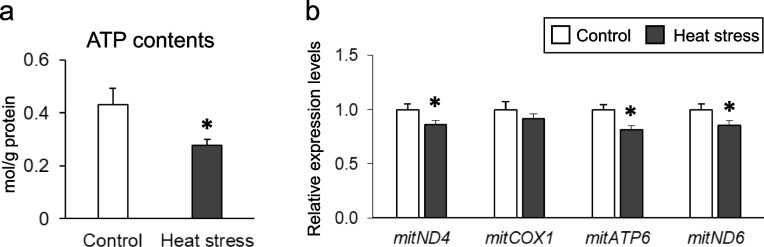
Effect of chronic heat stress on the functionality of mitochondria in the kidney. **a** ATP levels in renal tissue. *n* = 8 in each group. Data are presented as the percentage of the total cortex and as the mean ± SEM. Statistical analysis was performed using a Student’s *t*-test. **b** mtDNA copy number of renal tissues in laying hens. mt/nucDNA = mtDNA relative to nuclear DNA (β-actin) copy number. ^*^P < 0.05

As shown in Fig. 2a, chronic heat exposure significantly decreased ATP content of the renal tissue. Quantitative PCR analysis was used to analyze mitochondrial DNA copy number (mtDNA-CN) in the renal tissue of laying hens based on the mitochondrial *ND4, COX1, ATP6,* and *ND6* genes. The ratio of mt/nuclear DNA was significantly decreased for the *ND4*, *ATP6*, and *ND6* genes in heat-stressed birds (Fig. 2b).

### Effect of chronic heat stress on the expression of cGAS-STING pathway genes and related factors

**Fig. 3:**
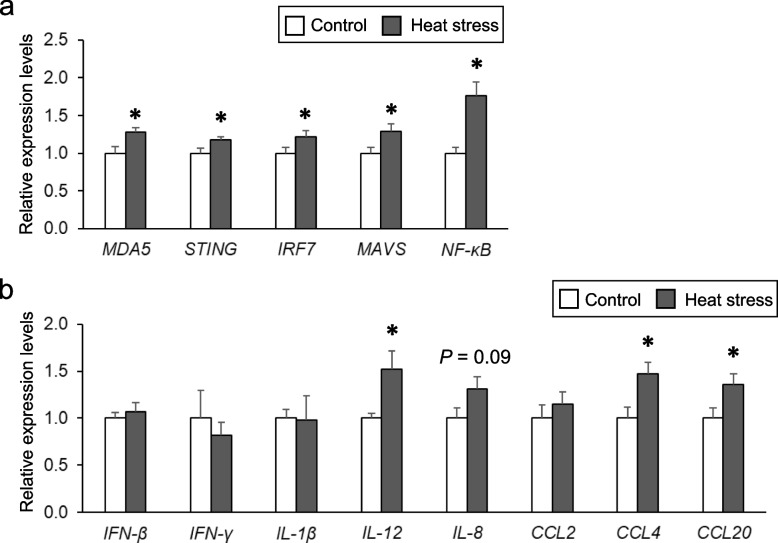
Effects of chronic heat stress on the expression of STING, NF-kB pathway, and related genes. **a** mRNA levels of *MDA5, STING, IRF7, MAVS,* and *NF-kB*, **b** mRNA levels of pro-inflammatory cytokines (*IFN-β, IFN-γ, IL-1β,* and *IL-12*) and chemokines (*IL-8, CCL2, CCL4* and *CCL20).* The mRNA expression levels were quantified by real-time quantitative PCR and normalized to 18S rRNA. *n* = 8 in each group. Statistical analysis was performed using a Student’s *t*-test. ^*^P < 0.05

As shown in Fig. 3a, the expression of the *MDA5, STING, IRF7,* and *MAVS* genes associated with the cGAS-STING pathway and the corresponding regulatory *NF-kB* gene were significantly increased in the heat-stressed group compared with that in the control group. Furthermore, the expression levels of pro-inflammatory cytokines (*IL-12*) and chemokines (*CCL4* and *CCL20*) were significantly upregulated, and the expression of *IL-8* tended to increase in heat-stressed hens (Fig. 3b). In contrast, the expression of other pro-inflammatory cytokines and chemokines (*IFN-β, IFN, IL-1β* and *CCL2*) was not altered by heat exposure (Fig. 3b). These results indicate that heat exposure stimulates the cGAS-STING pathway and subsequent inflammation.

## Discussion

Heat exposure is a nonspecific stressor that can affect the welfare of livestock and cause death [39]. In the present study, we focused on renal damage in hens during the laying period. Chronic heat stress in laying hens increased plasma creatinine and decreased albumin levels, indicating that chronic heat exposure causes renal failure. Furthermore, heat stress in laying hens significantly increased the renal cortical fibrotic area and the expression of pro-fibrotic genes. To our knowledge, this is the first report demonstrating that heat stress induces renal fibrosis in laying hens. The results suggest that renal failure and fibrosis are responsible, at least in part, for the reduction of laying performance and eggshell quality during conditions of heat stress.

Renal fibrosis is a common pathological feature of chronic renal injury [40, 41]. A recent study indicated that mitochondrial dysfunction is a major problem in the development and progression of renal fibrosis [42]. In addition, studies have provided evidence of a relationship between mitochondrial damage and dysregulated quality of mtDNA [43, 44]. mtDNA-CN refers to the abundance of mitochondria in a cell which depends on energy requirements and can be measured by mtDNA copy number and quality of mtDNA fragments [45]. Low tissue mtDNA-CN is associated with higher levels of oxidative stress and is a causative factor for oxidative stress-related damage [46]. Heat stress affects mitochondrial function and is characterized by increased levels of reactive oxygen species and an imbalance in the mitochondrial redox state [47]. Zhang et al. [48] showed that mtDNA-CN and ATP were decreased in the liver of broiler chickens subjected to chronic heat exposure. Similarly, our results demonstrate that mtDNA-CN in chronic heat-stressed hen kidneys was significantly reduced compared with that of the control group. Moreover, ATP content was also significantly lower in heat-stressed hen kidneys compared with that in the unexposed hens. Thus, chronic heat stress leads to severe mitochondrial damage in the kidneys of laying hens, which may contribute to the progression of renal fibrosis and dysfunction.

Recently, Maekawa et al. [26] concluded that mitochondrial dysfunction and activation of the mtDNA-cGAS-STING pathway are critical regulators of mammalian kidney injury. Cyclic guanosine monophosphate-adenosine monophosphate synthase (cGAS) has recently been identified as key cytosolic DNA signal that mediates type I IFN signaling in autoimmune diseases [49]. The leakage of DNA itself from the nucleus or mitochondria into the cytosol under conditions of stress or cellular injury can cleave cGAS which converts ATP and GTP to the second messenger cyclic GAMP which mediates the activation of STING [50]. To identify the underlying mechanisms of renal fibrosis in heat-stressed hens, we measured the expression of cGAS-STING pathway-related genes by qRT-PCR. We found that the expression of the *MDA5, STING, IRF7, MAVS* and *NF-κB* genes was increased in the heat-stressed group. Furthermore, the expression of pro-inflammatory cytokine (*IL-12*) and chemokine (*IL-8*, *CCL4*, and *CCL20*) genes was upregulated in heat-stressed hens. These results indicate that the activation of the mtDNA-cGAS-STING pathway and subsequent inflammation was induced in the kidneys of heat-stressed laying hens. This suggests that in the laying hens, similar to mammals, mtDNA is a key driver of inflammation and a cellular mechanism involved in the development of inflammation and renal dysfunction. In the present study, mRNA levels of pro-inflammatory cytokine and chemokines were upregulated by chronic heat stress in the kidneys of laying hens. Both pro-inflammatory cytokines and chemokines affect the onset and progression of renal disease [51]. IL-12 promotes renal injury through IFN-γ secretion and crescent formation in a mouse model of lymphocyte accumulation [52–54]. In addition, serum and renal IL-12 levels are increased in patients with kidney disease [55, 56]. Chemokines stimulate the migration of immune cells and increase the production and activity of adhesion molecules that contribute to fibrosis and kidney damage [36, 57]. In humans, increased expression of the *IL-8*, *CCL4*, and *CCL20* genes are involved in the pathogenesis of renal diseases [58–60]. Our data showing high levels of pro-inflammatory cytokine and chemokine gene expression in heat-stressed hens indicates that the release of these cytokines contributes to the progression of renal fibrosis and failure.

## Conclusions

Our results indicate that chronic heat exposure induces renal fibrosis and mitochondrial damage in laying hens. Mitochondrial damage by heat stress activates mtDNA-cGAS-STING signaling and subsequent inflammation, which contributes to the progression of renal fibrosis and dysfunction. Because renal mitochondrial damage induces renal fibrosis through activation of mtDNA-cGAS-STING pathway, mitochondria-targeting compounds or STING pathway inhibitors may represent a strategy to treat renal fibrosis and dysfunction after exposure to heat stress in laying hens.

## Data Availability

All data generated or analyzed during this study are available from the corresponding authors on reasonable request.

## Abbreviations

ATP6: ATP synthase F0 subunit 6
αSMA: α-Smooth muscle actin
CCL2: C–C motif chemokine ligands 2
CCL4: C–C motif chemokine ligands 4
CCL20: C–C motif chemokine ligands 20
cGAS: Cyclic GMP-AMP synthase
COL1A1: Collagen type I alpha 1
COL1A2: Collagen type I alpha 2
COX1: Mitochondrial cytochrome c oxidase 1
IFN-β: Type I interferon-β
IFN-γ: Type I interferon-γ
IL-1β: Interleukin-1β
IL-8: Interleukin-8
IRF7: Type I interferon regulatory factor 7
MAVS: Mitochondrial antiviral signaling
MDA5: Melanoma differentiation-associated gene 5
mtDNA-CN: Mitochondrial DNA copy number
ND4: NADH dehydrogenase subunit 4
ND6: NADH dehydrogenase subunit 6
STING: Stimulator of interferon genes
TGF-β: Transforming growth factor-β
18S rRNA: 18S ribosomal RNA
